# Correction: The effect of core stability training on ball-kicking velocity, sprint speed, and agility in adolescent male football players

**DOI:** 10.1371/journal.pone.0334020

**Published:** 2025-10-07

**Authors:** The PLOS One Staff

The affiliation for the second and third authors were swapped. Zehra Güçhan Topçu is not affiliated with #2 but with #3: Faculty of Health Sciences, Physical Therapy and Rehabilitation Department, Eastern Mediterranean University, Famagusta, Cyprus. Volga Bayrakcı Tunay is not affiliated with #3 but with #2: Faculty of Physical Therapy and Rehabilitation, University of Hacettepe, Ankara, Turkey

The publisher apologizes for the errors.
